# Retroperitoneal cavernous hemangioma: A case report with literature review

**DOI:** 10.1016/j.radcr.2025.10.049

**Published:** 2025-11-13

**Authors:** Omer H. Ghalib, Rawa Bapir, Hemin A. Hassan, Las L. Hussain, Deari A. Ismaeil, Soran H. Tahir, Kayhan A. Najar, Jihad I. Hama, Hiwa O. Abdullah, Fahmi H. Kakamad

**Affiliations:** a Scientific Affairs Department, Smart Health Tower, Madam Mitterrand Street, Sulaymaniyah, 46001, Iraq; b Department of Urology, Sulaymaniyah Surgical Teaching Hospital, Zanko Street, Sulaymaniyah, 46001, Iraq; c Kscien Organization for Scientific Research (Middle East Office), Hamdi Street, Azadi Mall, Sulaymaniyah, 46001, Iraq; d Department of Pathology, Hiwa Cancer Hospital, Shorsh Street, Sulaymaniyah, 46001, Iraq; e College of Medicine, University of Sulaimani, Madam Mitterrand Street, Sulaymaniyah, 46001, Iraq; f Research Center, University of Halabja, Ababaile Village, Zanko Street, Halabja, 46018, Iraq

**Keywords:** Retroperitoneal tumor, Cavernous hemangioma, Benign vascular tumor, Retroperitoneal neoplasm

## Abstract

Retroperitoneal cavernous hemangiomas (RCHs) are exceedingly rare benign vascular tumors. They pose significant diagnostic challenges due to their nonspecific clinical presentations and imaging findings. This report highlights a clinically misdiagnosed case of RCH as a different retroperitoneal tumor. A 43-year-old female presented with persistent right hypochondrial pain. Imaging studies suggested a retroperitoneal mass, initially suspected to be either a gastrointestinal stromal tumor (GIST) or Schwannoma, or paraganglioma. Surgical resection of the tumor was performed, and histopathological examination confirmed the diagnosis of a cavernous hemangioma. The patient recovered well with no postoperative complications. Limited cases of RCHs have been reported in the literature. These tumors often mimic other retroperitoneal masses such as GISTs. Imaging findings are nonspecific, and definitive diagnosis typically relies on histopathological analysis. Surgical resection is the mainstay of treatment, with excellent outcomes reported across cases.

## Background

Retroperitoneal tumors constitute a rare group of neoplasms, the majority of which are malignant; however, they account for less than 0.5% of all malignancies [1,2]. Among these, vascular tumors such as hemangiomas are particularly uncommon, comprising only 1-3% of retroperitoneal tumors [1]. Cavernous hemangiomas, a benign subtype of these vascular lesions, are most frequently observed in the liver, skin, and mucosal tissues. However, their occurrence in the retroperitoneum is exceedingly rare, making them a diagnostic challenge [3,4]. Since 1950, fewer than 30 cases of adult retroperitoneal hemangioma have been reported. These tumors most often develop in the kidney, pancreas, or adrenal glands. However, primary retroperitoneal cavernous hemangiomas (RCHs) are even rarer and unique for being completely separate from surrounding organs [5]. The clinical presentation of RCHs is often nonspecific, with symptoms like abdominal discomfort or mass effect, if present, being subtle and easily overlooked. Due to the overlapping features in radiology with other retroperitoneal masses, clinical diagnosis of RCHs is challenging [5]. On computed tomography (CT), RCHs appear as well-defined, low-attenuation masses with little or no contrast enhancement, often resembling cystic or other benign retroperitoneal lesions. Magnetic resonance imaging (MRI) typically shows low T1 and high T2 signal intensities, sometimes with heterogeneous postcontrast enhancement. These features can overlap with other retroperitoneal tumors such as gastrointestinal stromal tumors (GISTs), lymphangiomas, and liposarcomas, making histopathological examination after surgical resection necessary for definitive diagnosis [6]. This report highlights a clinically mistaken case of RCH as a different retroperitoneal tumor.

## Case presentation

*Patient information.* A 43-year-old female patient presented with right acute hypochondrial pain. She was diabetic and taking Metformin (500 mg, 1×3) without any other comorbidities. Her surgical history included 4 cesarean sections. Her family history was negative for any malignant disease.

*Clinical findings*. The patient was conscious and alert. There were no associated signs and symptoms, such as fever, jaundice, dark urine, pale stool, vomiting, or melena. Her vital signs and physical examinations were normal.

**Fig. 1:**
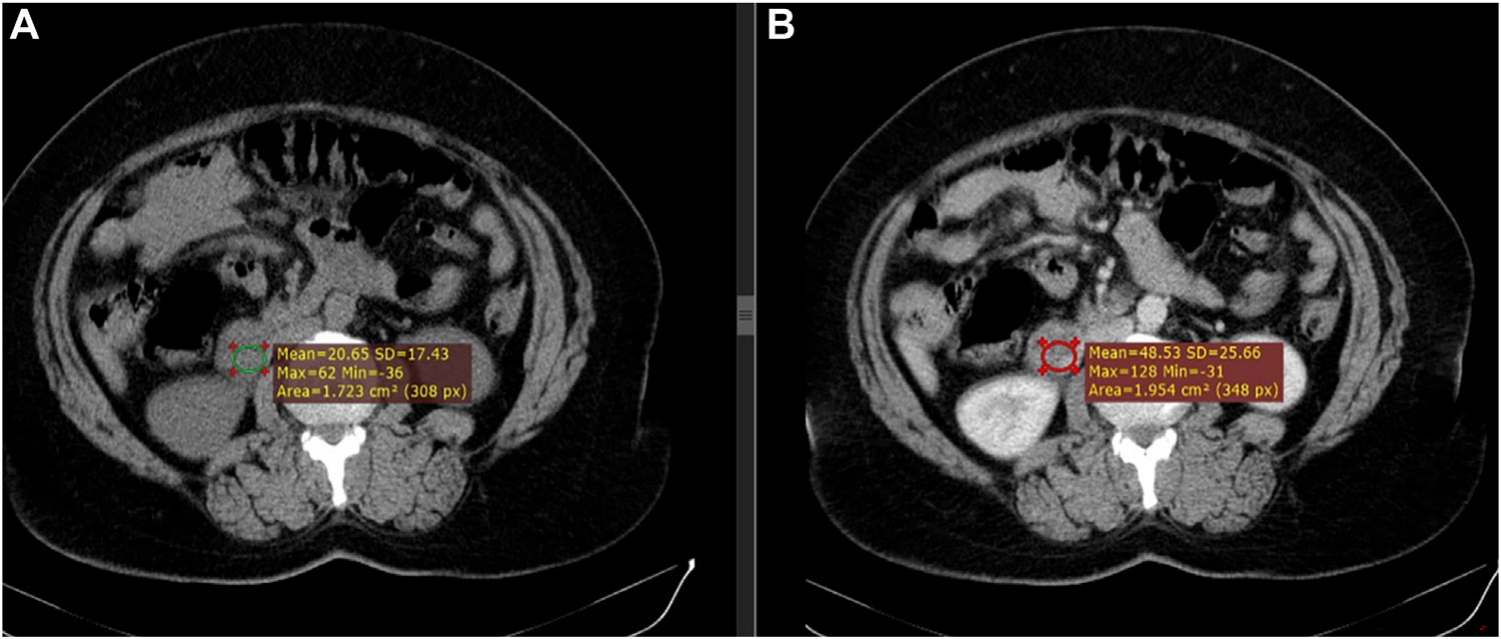
Contrast-enhanced CT images of the abdomen. Portal venous (green circle) and Excretory phases show a heterogeneously enhanced mass (red circle). Structures surrounding the lesions are: medially the inferior vena cava; posteriorly right gonadal vein and right ureter, anteromedially duodenum, anterolaterally mesenteric fat, laterally right kidney and ascending colon. The density of the lesion in the native scan is 20 HU, and in the postcontrast scan is 48HU.

**Fig. 2:**
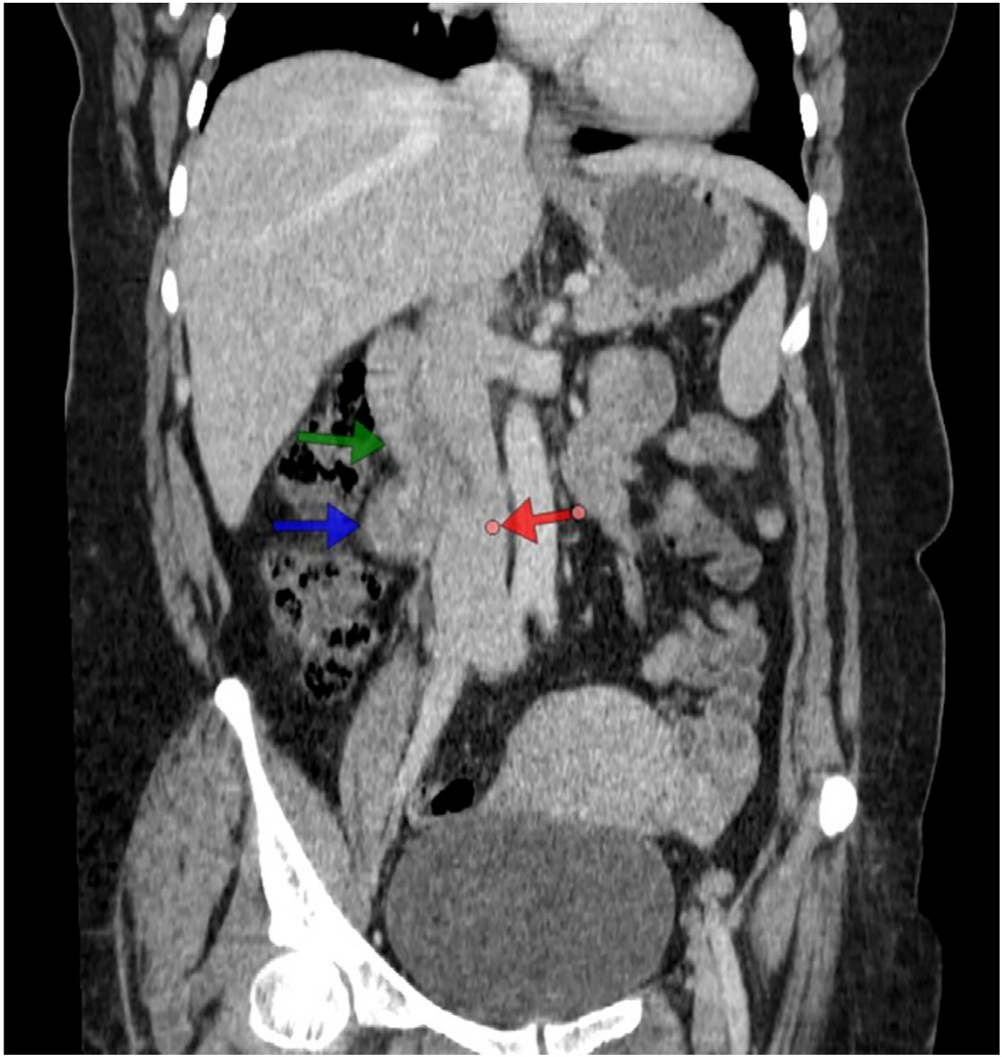
Contrast-enhanced CT of the abdomen, coronal section, shows a retroperitoneal enhanced mass “blue arrow”, the second part of the duodenum superior to the mass and in contact with it “green arrow”, and the IVC medial to the mass “red arrow”.

*Diagnostic assessments.* Blood investigations revealed normal complete blood count, renal function test, serum electrolytes, total serum bilirubin, liver function test, amylase, and lipase. C-reactive protein was elevated (40.2 mg/L, normal range: 0-5 mg/L). An abdominal ultrasound showed several gallstones and a normal thickness of the gallbladder wall along with normal biliary ducts. A contrast-enhanced CT scan of the chest, abdomen, and pelvis revealed a 4.5 × 3.4 × 2.8 cm avidly enhancing mass with a well-defined, lobulated margin in the right retroperitoneal region (Figs. 1 and 2). It had features of GIST, and retroperitoneal neurogenic tumors like Schwannoma and paraganglioma originating from the duodenum. Esophagogastroduodenoscopy revealed no lesions or masses, aside from erosions observed in the gastric body and antrum.

**Fig. 3:**
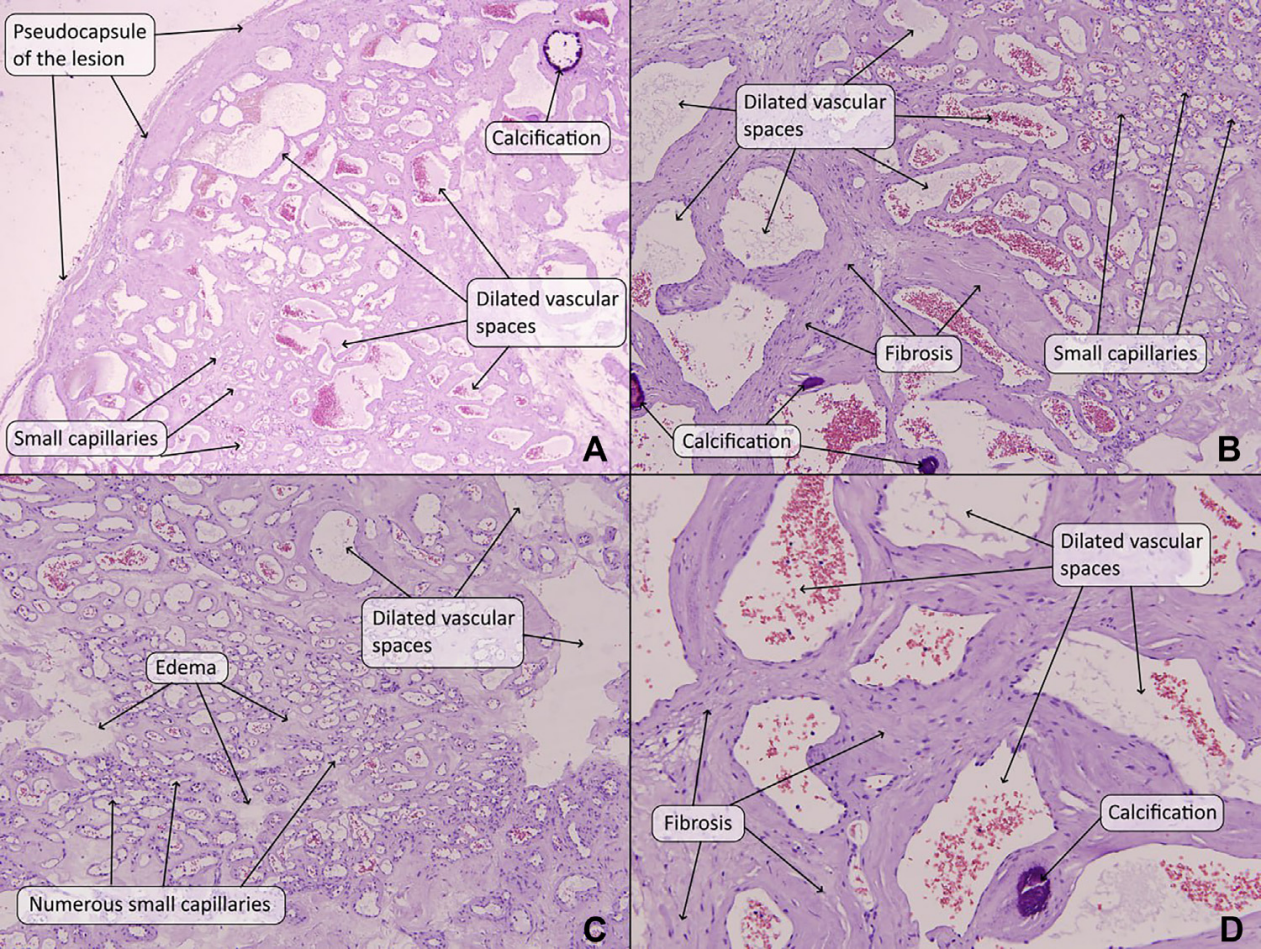
Histopathological examination of the mass. (A-D) The lesion is surrounded by a thin, fibrous pseudocapsule and is composed of an admixture of widely dilated, congested, and thin-walled vascular channels and small capillaries. There is intervening fibrosis, edema, and calcification. There is no atypia of the endothelial cell nuclei, significant mitotic activity, or necrosis. [Hematoxylin and eosin (A-D); original magnification x 40 (A), x 100 (B and C), x 400 (D)].

*Therapeutic intervention and follow-up***.** Under general anesthesia, with the patient in the supine position, a liver donor incision was made. A cholecystectomy was performed. Subsequently, Kocherization of the colon was done. Upon exploration, the mass was found to be nonadherent to the duodenum and excised. Histopathological examination of the mass was consistent with a cavernous hemangioma (Fig. 3). The postoperative period was uneventful, and the patient was discharged home without complications. At the 6-month postoperative follow-up, the patient was doing well and remained clinically stable. Abdominal ultrasound showed no abnormalities or evidence of recurrence.

## Discussion

In the present case, the references have been filtered out to avoid citing non-peer-reviewed data, and the case has been written according to CaReL guidelines [7,8]. The rarity of RCHs combined with nonspecific clinical presentations and overlapping imaging characteristics presents significant diagnostic challenges [4,5]. The disease may be identified incidentally during imaging studies performed for other reasons, while it may also present with vague complaints, such as abdominal pain or discomfort [1,3,5]. Moreover, RCHs may exhibit imaging features similar to those of anastomosing hemangioma, potentially mimicking its appearance [9]. A review of 5 documented cases, summarized in Table 1, demonstrates the variability in clinical and diagnostic features of RCHs. The age range of patients spanned from 35 to 73 years, with an almost equal gender distribution. Common symptoms included abdominal pain, as seen in 3 cases [1,5,10], abdominal distension in 2 cases [4,10], and 1 case was asymptomatic [3]. This diversity underscores the unpredictable clinical course of these tumors. The present case was a female who presented with right hypochondrial pain.

**Table 1:** Summary of clinical characteristics of 5 cases of retroperitoneal cavernous hemangioma.

Author [reference]	Country	Age (years)	Gender	Symptoms	Imaging findings	Size (cm)	Surgical approach
He et al. [1]	China	38	Male	Epigastric pain	Hypodense mass on CT	7.6	Open resection
Laih et al. [3]	Taiwan	57	Female	Incidental finding	Enhancing mass on CT	4.2	Open resection
Zielinski et al. [4]	Poland	71	Female	Abdominal distension, limb edema, dyspnea	Large retroperitoneal mass	18.3	Open resection
Debaibi et al. [5]	Tunisia	35	Male	Chronic abdominal pain	Hypodense mass on CT	4	Open resection
Matsui et al. [9]	Japan	73	Male	Chronic abdominal discomfort and distension	Nonenhanced mass on CT	35	Open resection

Imaging findings in RCHs vary significantly, often mimicking malignant or other vascular lesions. Contrast-enhanced CT and MRI are commonly employed for initial evaluation. The imaging demonstrates enhancing retroperitoneal masses in most cases, including the current case. These imaging characteristics overlap with other retroperitoneal tumors, such as GISTs or lymphangiomas, as seen in the report from Taiwan [3] and the current case. However, in the case report by Matsui et al., the tumor appeared as a nonenhancing mass on CT scan. This highlights the variability in the radiologic characteristics of the tumor, which makes the diagnosis more challenging [10]. Tumor size ranged widely, from 4.5 cm to a massive 20 cm, with larger lesions frequently causing symptoms due to compression of surrounding structures [4]. The tumor size among the reviewed cases ranged from 4 cm to 35 cm, with a mean size of 13.8 cm. Smaller lesions are often discovered incidentally on imaging, whereas larger tumors are more likely to become symptomatic due to mass effect or hemorrhage [11,12]. Surgical complexity also increases with tumor size; giant lesions frequently necessitate open approaches, meticulous vascular control, and, in some cases, preoperative planning with interventional radiology, while smaller or more accessible tumors may be amenable to laparoscopic management [[1], [11]].

Surgical resection is the treatment of choice for RCHs. A complete excision provides both diagnostic guidance and therapeutic benefits. Despite their benign nature, these tumors can cause significant complications due to mass effect or mimic malignancies, necessitating removal. All reviewed cases, along with the present case, underwent open surgical resection, with no recurrences observed during follow-up. This favorable prognosis highlights the efficacy of surgical management [3,4,10]. Notably, larger tumors may require meticulous surgical planning due to their size and proximity to vital structures [1,4].

Histopathological examination remains the gold standard for confirming RCHs. The hallmark features include large, thin-walled vascular spaces lined with flattened endothelial cells. Immunohistochemical markers, such as CD31 and CD34, consistently confirm the vascular origin of these tumors [1,5]. This differentiation is critical, as RCHs can mimic malignant vascular lesions. In the present case, histopathological analysis following surgical excision confirmed the diagnosis of a cavernous hemangioma, aligning with findings reported in the literature.

## Conclusion

Because of their variable imaging characteristics, RCHs may be misdiagnosed as other retroperitoneal tumors. Surgical resection can be considered as a potential treatment option, with histopathological examination possibly serving as the definitive means of diagnosis.

## Patient consent

Consent has been taken from the patient.

## Authors contribution

OHG and RB were major contributors to the conception of the study, as well as to the literature search for related studies and managed the case and LLH, DAI, and JIH were involved in the literature review, the design of the study, the critical revision of the manuscript and the processing of the figures and HAH was the pathologist who performed the histopathological diagnosis and SHT was the radiologist who assessed the case and KAN, HOA, and FHK were involved in the literature review, study design, and in the writing the manuscript. All authors have read and approved the final manuscript.
